# Evaluation of an intervention to promote minimally invasive dentistry (MID) in an Australian community dental agency—A pilot study

**DOI:** 10.1111/idh.12523

**Published:** 2021-06-02

**Authors:** Tan Minh Nguyen, Utsana Tonmukayakul, Hanny Calache

**Affiliations:** ^1^ Deakin Health Economics Institute for Health Transformation Deakin University Waurn Ponds Vic. Australia; ^2^ Community Dental Program Peninsula Health Frankston Vic. Australia; ^3^ Dentistry and Oral Health La Trobe Rural Health School La Trobe University Bendigo Vic. Australia

**Keywords:** dental care for children, dental caries, dental health services, education, dental, continuing, fluorides, topical, quality assurance, health care

## Abstract

**Objectives:**

To evaluate the impact of an intervention consisting of a 1‐day continuing professional development (CPD) education programme on the International Caries Classification and Management System (ICCMS^™^), and monthly performance feedback, and to promote minimally invasive dentistry (MID) for children aged under 12 years in an Australian community dental agency. The *a priori* hypotheses assumed the intervention would increase preventive services, and treatment demand was met.

**Methods:**

A quasi non‐randomized controlled trial with convenience sampling method was adopted. Fourteen dental practitioners received the intervention. The prevalence of dental caries and gingivitis in Australian children was used to determine the treatment demand and used as the performance benchmark. Ten types of preventive and non‐preventive dental services were examined. A Difference‐in‐Differences (DiD) of 12‐month pre‐ (baseline) and post‐intervention analysis was performed.

**Results:**

The intervention group demonstrated increases in topical fluoride application and dietary analysis and advice services. The standard care group had increases in oral prophylaxis or scale and clean, topical fluoride application and oral hygiene instructions (*p*‐value <0.05). The DiD analysis confirmed the above findings in the intervention group, while other preventive services declined. In the intervention group, the performance benchmark for oral prophylaxis or scale and clean and oral hygiene instructions was met at baseline and post‐intervention.

**Conclusions:**

Only a few preventive services had already met the performance benchmark. The intervention was associated with varied changes to preventive and non‐preventive dental services. More robust study design addressing the study limitations and validating the performance benchmark is required.

## 1 INTRODUCTION

Dental caries management is dominated by a treatment‐focussed approach and has not reduced the prevalence of oral diseases.^1^ Globally, the burden of untreated dental caries has remained relatively unchanged over the past 30 years, despite significant advances in clinical techniques and dental technology.^2^ In Australia, dental conditions are the second most common cause of acute potentially preventable hospitalizations, particularly among children; this is an expensive approach for oral health care.^3^

Australian oral health surveillance data suggest that children under five years of age do not receive care early or frequently enough nor do they receive sufficient levels of preventive dental care.^4^ The importance of early dental caries detection and prevention among Australian children addresses the key action areas in Australia's National Oral Health Plan 2015–2024.^5^ They include:

1. Extending access to the preventive effects of fluoride,
2. Encouraging participation in clinical audit and benchmarking programmes,
3. Enhancing skills and competencies within the oral health workforce to meet the needs of priority populations, and
4. Supporting research that develops and evaluates oral health promotion programmes, models of oral health care and access to care for priority populations.

The International Caries Classification and Management System (ICCMS™) is an internationally developed evidence‐based guideline for the assessment and management of dental caries.^6^, ^7^, ^8^ It incorporates the collection of individual risk and protective factors, as well as diagnostic and clinical indicators. This information informs a dental caries management plan that ranges from preventive to surgical interventions. Comprehensive detail of the ICCMS™ approach was published in 2014.^6^ It is supported by the *Federation Dentaire Internationale* and is based on the minimally invasive dentistry (MID) concept.^8^

Although the ICCMS™ is progressively integrated within Australian and New Zealand dental education curricula,^9^ its application in practice is limited. One possible reason is the ICCMS™ is new, so dental practitioners may not know or may not have received training on the ICCMS™. Another possible explanation would relate to the uptake of the MID concept itself. In Australia, the MID philosophy has not been fully adopted in public dental services.^10^ A systematic review and meta‐analysis found that dental practitioners are more likely to intervene surgically when best practice requires less invasive treatment.^11^ One strategy that can promote the adoption of the ICCMS™ among dental practitioners who have not been exposed to this guideline is a capacity building programme through continuing professional development (CPD) activities.^12^, ^13^, ^14^, ^15^ Capacity building training programmes on ICCMS™ have not been officially provided within community dental programme for dental practitioners working in the Victorian public sector, Australia.

This pilot study aimed to evaluate an intervention promoting the MID approach in an Australian community dental agency. The intervention consisted of a 1‐day ICCMS™ training as a CPD activity and monthly performance feedback against a developed performance benchmark. Two *a priori* hypotheses assumed selected preventive dental services would increase for the intervention group over 12‐month post‐intervention, and treatment demand for these preventive services would be met. The project objectives were to.

1. Report on the type and rates of preventive, restorative and extraction procedures provided 12 months before the intervention and 12‐month post‐intervention for children 0–12 years of age
2. Apply the Difference‐In‐Difference (DiD) analysis to control for bias and make inferences on the impact of the intervention.
3. Report on the type and rates of the different dental procedures provided to children 0–12 years of age against the developed performance benchmarks

## 2 STUDY POPULATION AND METHODOLOGY

A quasi non‐randomized controlled trial with convenience sampling method was adopted. Four dental services sites within the community dental agency were included in this study. In the intervention group, 14 dental practitioners were recruited from the two dental service sites with a skill mix profile of 4.8 full‐time equivalent (FTE) dentists and 3.2 FTE dental/oral health therapists. The other remaining 15 dental practitioners working at the other two dental service sites were denoted as the standard care group (control) with a skill mix of 5.9 FTE dentists and 4.2 FTE dental/oral health therapists. Both groups had a dentist to dental/oral health therapist ratio of approximately 1.5:1. Participating dental practitioners at times are required to work ‘cross‐sites’ within the community dental agency.

Dental practitioners in the intervention group received a 1‐day CPD programme on the ICCMS™ in July 2017 delivered by the two researchers (TMN and HC), whereas dental practitioners in the standard care group did not. The topics covered in the 1‐day CPD programme included the following:

- Rationale and principles for ICCMS™
- Theory of ICCMS™
- Clinical protocol of ICCMS™
- Case study examples of ICCMS™
- Enablers and barriers to ICCMS™

The types of dental services provided by the community dental agency were classified into:

1. Preventive Dental Services (routine oral examination, topical fluoride application, bitewing intra‐oral radiographs, dietary analysis and advice, oral hygiene instructions, oral prophylaxis or scale and clean and fissure sealants), and
2. Non‐Preventive Dental Services (posterior restorations, either tooth coloured (conventional) or using preformed metal stainless steel crowns (SSC) and dental extractions).

Since there is no formal performance benchmark for preventive dental services, the research team in consultation with the organization's leadership dental team developed one based on the prevalence of dental caries and gingivitis for Australian children aged 0–12, consistent with the ICCMS^TM^ and MID approach.^15^ (Refer to Appendix 1 for a full description and evidence‐based justifications). Dental practitioners in both groups can develop individualized dental treatment plans and provide dental treatment based on their clinical judgement.

**FIGURE 1:**
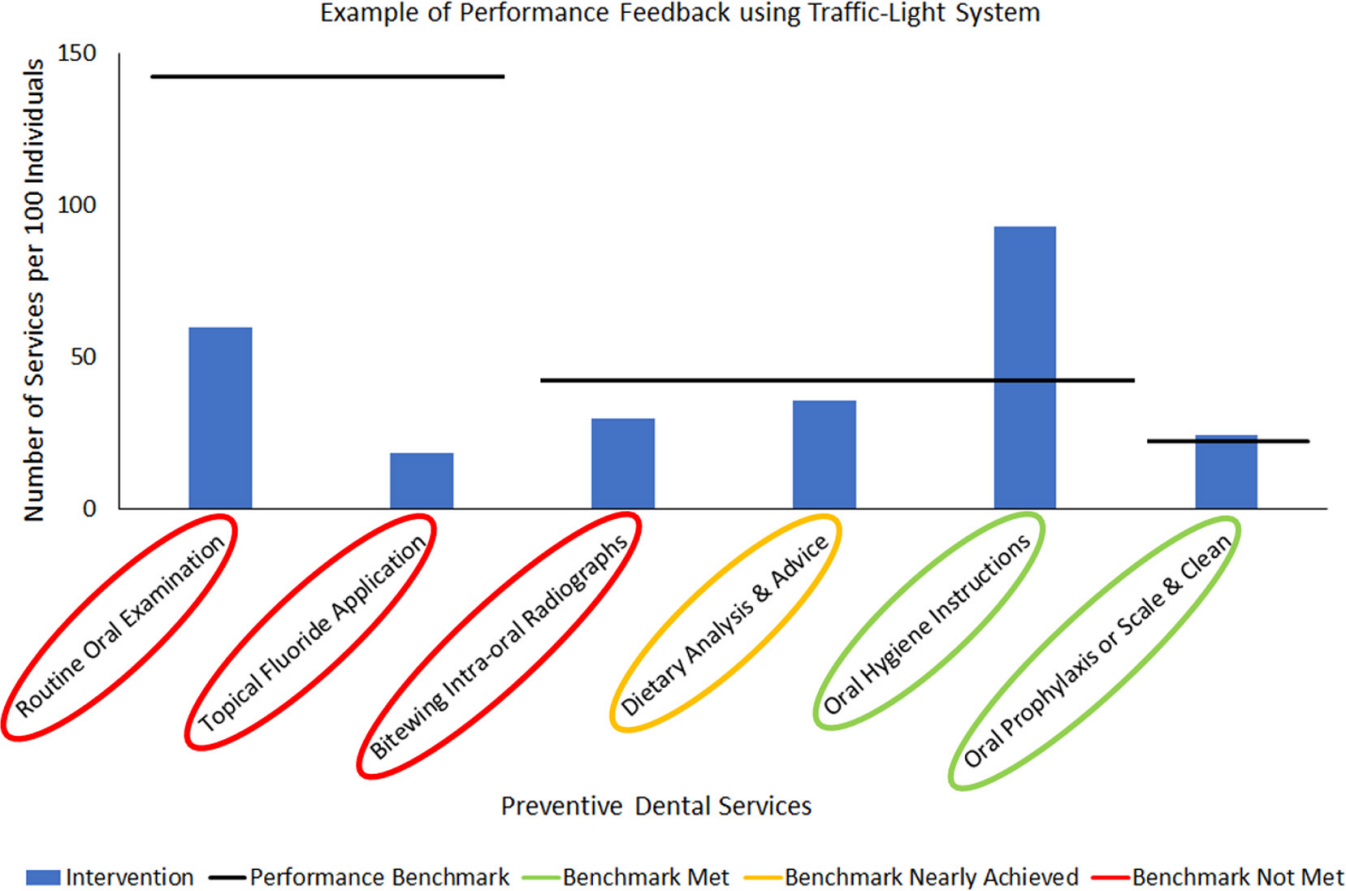
An example of performance feedback using the traffic light system. [Colour figure can be viewed at wileyonlinelibrary.com]

Monthly performance feedback based on dental services provided in the last 12 months by the intervention group was summarized based on the classified category of the type of service and compared against the performance benchmark. Feedback on how well the intervention group performed, using a traffic light reporting system, was presented to the participating dental practitioners by the senior team leader at regular monthly team meetings. Figure 1 provides an example of the monthly feedback report.

The traffic light reporting system was adapted from a clinical education intervention reported in the literature and has been shown to improve the behaviour of undergraduate dental students.^16^ A rapid literature review reported that feedback interventions supporting individuals, teams, and organizations have value in reducing unwarranted clinical variation.^17^

De‐identified dental services item codes provided in dental clinics and school‐based dental services for children aged 0–12 years were obtained for dental services provided for baseline (pre‐intervention) and 12‐month post‐intervention analysis for the 2016/17 and 2017/18 financial years. Two‐sample proportions *z*‐tests determined whether there was a statistically significant difference (*p* < 0.05) for dental treatment services received by children provided by dental practitioners in the intervention and standard care groups.

The magnitude of the intervention effect was calculated using the DiD approach. The DiD study design can make inferences where randomized controlled trials are impractical, infeasible or unethical.^18^ Under the DiD study design, characteristics of sample size in the intervention and control group do not need to be similar.^19^ The flexible features of the DiD approach make it appropriate to evaluate the intervention in this pilot study.

The DiD approach is commonly used for studies, which involve data collected for other purposes such as information from electronic health records or medical claims datasets. Essentially, it is a before‐after comparison test of an intervention group against a comparator group with similar characteristics and exposure to potential confounders.

To calculate the DiD, the effect on the intervention group is denoted I^Pre^, and I^Post^ for before and after, respectively. Likewise, the comparator group is denoted, C^Pre^ and C^Post^ for before and after, respectively. The DiD estimate is derived as (I^Pre^ ‐ I^Post^)‐ (C^Pre^ ‐ C^Post^), which determines the magnitude of the effect and the direction of the effect, that is a positive value indicates an increase, a negative value indicates a decrease, and a zero value indicate no difference.

Microsoft Excel 365 (Microsoft Corporation) and Stata 12 IC (StataCorp) was used to manage and analyse the data. This study received approval as a quality assurance project QA/18/PH/4 from Peninsula Health and completed according to the principles of the Declaration of Helsinki.

## 3 RESULTS

At baseline, the total number of children aged 0–12 years was 3265 in the intervention group and 4973 in the standard care group. At 12‐month post‐intervention, there were 2853 in the intervention group and 4698 in the standard care group. Table 1 outlines the dental service type and the number of dental services provided per 100 individuals at baseline and 12 months together with *z*‐tests comparing children who received dental services by dental practitioners in the intervention and standard care groups.

**TABLE 1:** The number of dental services provided per 100 individuals in the intervention and standard care group at baseline and 12‐month post‐intervention.

Service type	Intervention			*p*‐value	Standard Care			*p*‐value
	Baseline	12 Months	∆		Baseline	12 Months	∆	
Routine oral examination	67.5	59.8	‐	<0.001*	56.1	53.6	‐	0.016*
Topical fluoride application	7.2	18.4	+	<0.001*	1.4	2.5	+	<0.001*
Bitewing Intra‐oral radiographs	32.7	29.4	‐	0.005*	18.9	20.0	+	0.147
Dietary analysis and advice	26.6	35.4	+	<0.001*	41.9	43.3	+	0.168
Oral hygiene instructions	97.8	93.0	‐	<0.001*	58.6	62.6	+	<0.001*
Oral prophylaxis or scale and clean	28.4	23.9	‐	<0.001*	14.5	16.3	+	0.012*
Fissure sealants	90.6	84.5	‐	<0.001*	68.6	67.8	‐	0.425
Posterior conventional restorations	32.1	22.4	‐	<0.001*	36.4	23.6	‐	<0.001*
Stainless steel crowns	8.1	12.4	+	<0.001*	5.3	13.8	+	<0.001*
Dental extractions	11.8	5.3	‐	<0.001*	11.0	5.1	‐	<0.001*

**∆** change in services; ‐ decrease in services; _+_ increase in services.

* Statistically significant.

The intervention group received more dental services in almost all service types than the standard care group. The intervention group demonstrated a significant increase in topical fluoride application and dietary analysis and advice services, while other preventive dental services were significantly reduced. Most preventive dental services in the standard care group increased, but only oral prophylaxis or scale and clean, topical fluoride applications and oral hygiene instructions services were statistically significant (*p* < 0.05).

A statistically significant decrease in non‐preventive services (ie posterior conventional restoration and dental extractions) was noted in both groups. The provision of SSC significantly increased by a fold of 1.5 and 2.6 in the intervention and standard care group, respectively. The magnitudes of the changes are shown in Table 2. The DiD approach demonstrated that the intervention group provided a substantially increased number of topical fluoride application and dietary analysis and advice services compared to the standard care group. However, a reduction in routine oral examination, bite intra‐oral radiographs, oral prophylaxis or scale and clean, oral hygiene instructions and fissure sealants were also observed.

**TABLE 2:** Differences in the number of dental services provided per 100 individuals between the intervention and standard care group and the DiD analysis by service type.

Service type	Baseline	12 Months	Difference‐in differences
Routine oral examination	11.4	6.1	−5.3
Topical fluoride application	5.7	15.8	10.1
Bitewing intra‐oral radiographs	13.8	9.3	−4.5
Dietary analysis and advice	−15.2	−7.8	7.4
Oral hygiene instructions	39.3	30.4	−8.9
Oral prophylaxis or scale and clean	13.9	7.6	−6.3
Fissure sealants	22.1	16.8	−5.3
Posterior conventional restorations	−4.3	−1.2	3.1
Stainless steel crowns	2.7	−1.4	−4.1
Dental extractions	0.8	0.2	−0.6

The performance benchmark changed from being unmet at baseline and met at 12‐month post‐intervention for dietary analysis and advice in the standard care group, while other services maintained their performance status (Table 3). Preventive dental services that met the performance benchmark at baseline and post‐intervention were oral prophylaxis or scale and clean in the intervention group, and oral hygiene instructions in both groups.

**TABLE 3:** Evaluating whether the performance benchmark for preventive dental services provided per 100 individuals was met in the intervention and standard care groups at baseline and 12‐month post‐intervention.

Service type	Benchmark	Intervention		Standard care	
		Baseline	12 Months	Baseline	12 Months
Routine oral examination	142	No	No	No	No
Topical fluoride application	142	No	No	No	No
Bitewing intra‐oral radiographs	42	No	No	No	No
Dietary analysis and advice	42	No	No	No	Yes
Oral hygiene instructions	42	Yes	Yes	Yes	Yes
Oral prophylaxis or scale and clean	22	Yes	Yes	No	No

Yes: benchmark met; No: benchmark not met.

## 4 DISCUSSION

The first *a priori* hypothesis for this study was to test if the intervention would lead to an increase in the provision of preventive dental services aligned with the ICCMS^TM^. However, our findings demonstrated that the intervention group was associated with a significant increase in only a few types of preventive dental services, specifically, topical fluoride application and dietary analysis and advice services. The provision of the other five preventive dental services had significantly decreased.

We expected a decrease in the number of posterior conventional restorations and dental extractions as a result of providing more early detection and preventive dental services. Significant reduction in these services was found in both groups (Table 1), which is in contrast, to a statistical increase in SSC services. The placement of SSC refers to both conventional procedures requiring significant reduction of tooth structure and the minimally invasive Hall Crown Technique restoration (HCT) which aims to seal dental caries with a SSC without tooth preparation, caries removal or the use of local anaesthesia.^22^ The HCT is aligned with the ICCMS^™^ approach, but differentiating the type of technique cannot be quantified since the dental item code was not available for HCT during the study period.

Before the commencement of this study, all dental practitioners at Peninsula Health had a CPD programme on HCT. It appears the HCT was well adopted, particularly by dental practitioners in the intervention groups as reflected in the higher SSC services at baseline compared to the standard care group (8.1 vs 5.3 per 100 individuals). The uptake for providing more SSC services continued to increase at 12 months as evidenced by significant differences for both groups compared to baseline (*p* < 0.05).

This study found that there were only a few types of preventive dental services provided in the intervention and standard care group that met the developed performance benchmark. A specific focus is required to increase the substantial deficit for the number of services provided for routine oral examination and topical fluoride application services. Our findings support future research is required to facilitate the adoption and adherence to the ICCMS^™^ and MID approach, which is person‐centred centred and emphasises regular preventive maintenance.^10^

The DiD calculation showed the intervention was only associated with a substantial increase of topical fluoride application and dietary analysis and advice services. Although an unfavourable direction for the oral prophylaxis or scaling and clean and oral hygiene instructions was noted from the DiD analysis, these preventive services had already met the performance benchmark at baseline of 22.0 and 42.0 per 100 individuals, respectively.

The improvement in the delivery of only some preventive dental services could potentially be associated with the current behaviour of dental practitioners and the Victorian public funding system. Two government reports reported the lack of prevention and early intervention services in the management of oral disease provided by Australian public dental programmes.^20^, ^21^ Both reports highlight that the fee‐for‐service funding model financially incentivizes treatment services rather than preventive services. Approximately 10% of the funding resources for Victorian public dental programmes were directed towards preventive interventions.^20^

Other possible contributing factors, such as the attitudes and beliefs of the dental practitioners on oral disease management, and the perceptions of the clients regarding preventive and treatment services, were not controlled nor collected in this study. A qualitative research study has since been conducted to identify those contributing factors and explore the reasons for the increase in only a few types of preventive dental services found in this quantitative analysis. A separate publication on the qualitative study is forthcoming.

There are several limitations of this study that may have impacted the results. A fundamental limitation is that the researchers did not control for the potential confounder that dental practitioners in both groups may have received previous education and training on the ICCMS™ and MID approach or had undertaken them during the study period.

The positive differences for all preventive dental services except for dietary analysis and advice services at baseline and 12‐month post‐intervention indicates dental practitioners in the intervention group may be more aligned to the ICCMS™ and MID approach. The true results of the intervention may also have affected if there was contamination due to dental practitioners working cross‐sites. Furthermore, since there were data limitations where aggregate information was provided and not of individual observations, testing for sensitivity and falsification using the DiD analysis approach was not possible.

Although the sample size was adequate at the dental service site level, the study was confined to one community dental agency in the Melbourne metropolitan area, which limits the generalizability of the results. Furthermore, robust study design and more extensive research to explore the enablers and barriers to the ICCMS^TM^ and MID approach, and validating the developed performance benchmark would be needed to support the work of this pilot study.

A strength of this study is it shows that the 1‐day CPD training together with regular performance feedback could tentatively be beneficial to increase more preventive dental services, particularly in topical fluoride application and dietary analysis and advice services. Given dental practitioners in the intervention group provides dental services for all ages, it would be interesting to observe if there has been any impact from the intervention with clients aged older than 12 years.

Online education and training could be established to minimize the cost of the intervention, thereby making access to the CPD training flexible and increase the uptake of the ICCMS^TM^ and MID approach by other dental practitioners. Previous work has shown system‐level strategies, including multimethod/multiphased CPD,^14^ and dental staff faculty training promoting professionalism can encourage positive changes of behaviour in dentistry.^23^ Additional qualitative and quantitative evidence is needed to support the efficacy and applicability of the intervention in other settings. We would also need to test the developed proposed performance benchmarks with a range of stakeholders to determine their appropriateness for promoting the ICCMS^™^ and MID approach.

## 5 CONCLUSION

The 1‐day CPD education programme on ICCMS™ together with monthly performance feedback could promote the MID approach, particularly concerning topical fluoride application and dietary analysis and advice services for children aged 0–12 in an Australian community dental agency. However, the DiD analysis and comparison against the developed performance benchmarks showed the delivery of routine oral examination, bitewing intra‐oral radiographs, and topical fluoride applications required improvement. Additional research at a larger scale is needed, within the community dental agency and with other dental service providers, to explore the benefits of the ICCMS^TM^ education training as well as assessing the appropriateness of the developed performance benchmarks.

## 6 CLINICAL RELEVANCE

### 6.1 Scientific rationale for study

There is limited quantitative evidence from the Australian public dental sector, demonstrating whether dental services provided are consistent with the MID approach.

### 6.2 Principal findings

This research demonstrated a 1‐day CPD education programme, and monthly performance feedback provided to dental practitioners was associated with positive changes in a few preventive dental services evaluated at 12‐month post‐intervention.

### 6.3 Practical implications

More research is needed to investigate how to support dental practitioners to promote the ICCMS^TM^ and the MID approach.

## CONFLICT OF INTEREST

Mr Nguyen, Dr Tonmukayakul and Professor Calache have nothing to disclose.

## AUTHOR CONTRIBUTION

T.M.N, U.T. and H.C. conceived the ideas; T.M.N. analysed the data and led the writing; T.M.N., U.T. and H.C. interpreted the results, contributed and finalized the critical intellectual content.

## Data Availability

Data available on request due to privacy/ethical restriction.

## APPENDIX 1

The proposed annual performance benchmark was used for selected preventive dental services. It was developed from the prevalence of dental caries and periodontal disease of Australian children aged 5–10 years old but applied for children under age 12 years old. It is worth noting there is an absence of epidemiological data for children aged under 4. According to the Australian National Child Oral Health Survey 2012–14, it was reported that 42% of children aged 5–10 years had a history of dental caries experience in the primary dentition, and 22% of children aged 5–14 years had gingivitis.^15^ For the permanent dentition, the dental caries experience ranged from 9% to 38% for children aged 6–8 years and 12–24 years, respectively.^15^ After consultation with the senior team leaders at the participating community dental agency, it was agreed that 42% of children between aged 0–12 years should be seen twice a year with biannual topical fluoridation applications. The remaining 58% of children would be seen once a year. This equates to the performance benchmark for routine oral examinations and topical fluoride applications being set at 142.0 per 100 individuals. The performance benchmark for bitewing intra‐oral radiographs, dietary analysis and advice and oral hygiene instructions was set at a rate of 42.0 per 100 individuals, for the same reason noted above. The performance benchmark for managing 22% of children who have gingivitis was set at 22.0 per 100 individuals for oral prophylaxis or scale and clean.

The performance benchmark was not applied for fissure sealants, restorative and extraction services since they cannot be informed by epidemiological data. Therefore, the clinical indication to recommend fissure sealants is not possible. As a result, the performance benchmark for fissure sealants, posterior conventional restorations, restorations using preformed metal stainless steel crowns and dental extractions was not developed. Dental item codes for each type of service were outlined as follows:

Routine oral examinations—includes item code 011—Comprehensive oral examination and 012—Periodic oral examination.

Topical fluoride application—includes item codes 121—Topical application of remineralization and/or charismatic agents, one treatment and 123—Concentrated remineralization and/or aerostatic agents, application—single tooth. Item code 123 assumed to be claimed twice per individual and converted as one unit of 121.

Bitewing intra‐oral radiographs—includes item code 022—Intra‐oral periapical or bitewing radiograph—per exposure. Assumes one pair of bitewing intra‐oral radiographs.

Dietary analysis and advice—includes item code 131—Dietary analysis and advice.

Oral hygiene instructions—includes item code 141—Oral hygiene instruction.

Oral prophylaxis or scale and clean—includes item codes 111—Removal of plaque and/or stain and 114—Removal of calculus—first visit.

Fissure sealants—includes item code 161—Fissure and/or tooth surface sealing—per tooth (first 4 services on a day) and 162—Fissure and/or tooth surface sealing—per tooth (after 4 occasions of 161 on a day).

Posterior conventional restorations—includes item codes 531–535—Composite resin restoration—(one, two, three, four and five) surface—posterior tooth—direct.

Stainless steel crowns—includes item code 576—Metallic crown—preformed, 586—Crown metallic—with tooth preparation—preformed and 587—Crown metallic—minimal tooth preparation—preformed (Hall crown). Note that item codes 586 and 587 was only recently developed during the intervention.

Dental extractions—includes item code 311—Removal of a tooth or part(s) thereof and 314—Sectional removal of a tooth or part(s) thereof.
